# The role of policy actors’ belief systems and interests in framing public health nutrition problems: a case study of obesity in Australia

**DOI:** 10.1017/S1368980025100517

**Published:** 2025-06-03

**Authors:** Patricia Ribeiro de Melo, Phillip Baker, Priscila Pereira Machado, Elly Howse, Tanita Northcott, Mark Lawrence

**Affiliations:** 1 School of Exercise and Nutrition Sciences, Deakin University, Wadawurrung, Geelong, Victoria, Australia; 2 The University of Sydney, School of Public Health, Gadigal, Sydney, NSW, Australia; 3 Institute for Physical Activity and Nutrition, Deakin University, Wadawurrung, Geelong, Victoria, Australia; 4 NSW Government, Gadigal, Sydney, NSW, Australia

**Keywords:** Food and nutrition policy, Belief systems and interests, Frame analysis, Obesity

## Abstract

**Objective:**

This study investigated how the belief systems and interests of policy actors shaped their framing of the causes and solutions to obesity and how this influenced policy recommendations.

**Design:**

Submissions to the Select Committee on Obesity Epidemic in Australia (SCOEA) were collected, and actors were classified according to their interests in commercial and non-commercial groups. A framework grounded in social constructionism was used to code frames and underlying belief systems. The SCOEA report was analysed to identify the representative distribution of belief systems in recommendations.

**Setting:**

Australia.

**Participants:**

None.

**Results:**

150 submissions were collected and analysed. 120 submitters were actors with non-commercial interests, including governments (*n* 13), non-government organisations (*n* 49), civil society groups and citizens (*n* 24) and academia (*n* 34). Thirty submitters were actors with commercial interests including food industry representatives (*n* 23) and health enterprises (*n* 7). Conflicting belief systems in the framing of obesity were identified among policy actors, particularly between commercial and non-commercial groups. Non-commercial actors framed obesity in biomedical, lifestyle and socio-ecological terms, whereas commercial actors exclusively framed obesity as an issue of individual choices and proposed behavioural change interventions. A broad range of belief systems expressed by the submitters was represented in the SCOEA final report.

**Conclusion:**

These findings illustrate how policy actors’ beliefs and interests shaped their frames and influenced the development of a key policy report. Policymakers seeking to advance obesity prevention policy must critically evaluate strategic framing by various actors and ensure that policy decisions are evidence-based and aligned with health, equity and ecological perspectives.

Over the past four decades, a rapid rise in obesity has been observed in all countries^(1)^. Currently, obesity is estimated to affect over one billion people globally and is associated with around 5 million deaths annually^(2)^. Although there have been some improvements, such as stabilising childhood obesity rates in certain regions^(3)^, only a few countries have been able to reverse or halt its rise^(4,5)^. The rising prevalence of obesity is driven by a multitude of causes, including systemic drivers like political and economic systems, environmental drivers, such as the built environment, as well as individual-level factors, like genetics and behavioural patterns^(6)^. However, dietary shifts facilitated by modern food systems, including an increase in ultra-processed foods and a decline in minimally processed foods, are regarded as the main drivers of this pandemic^(6,7)^.

Australia has one of the highest obesity rates globally, with an estimated 14 million people classified as overweight or obese^(8)^. In response, the Australian government has initiated several programmes aimed at preventing the rising prevalence of obesity^(9)^. These include the Healthy Food Partnership, a public–private collaboration aimed at promoting healthy eating; the National Obesity Strategy 2022–2032, a 10-year framework for tackling overweight and obesity; and the Select Committee into Obesity Epidemic (SCOEA), established by the Australian Senate to enquire into potential causes and most effective solutions to obesity^(10)^. Through extensive consultations with individuals and organisations, the SCOEA produced a final report containing actionable recommendations for policymakers to halt obesity^(11)^.

Food and nutrition policymaking, and the science that underpins it, is inherently political and significantly influenced by the competing beliefs and interests of policy actors^(12)^. In this study, ‘belief system’ refers to a set of values, ideas and assumptions that inform how policy actors interpret the causes of, and solutions to, obesity. This definition draws on Sabatier’s Advocacy Coalition Framework, which distinguishes between ‘deep core beliefs’ (broad ontological and normative values that are not specific to a policy arena), ‘policy core beliefs’ (beliefs about the magnitude, causes and consequences of a policy problem) and ‘secondary beliefs’ (views about appropriate policy instruments and strategies to achieve goals aligned with policy core beliefs)^(13)^. This study focuses on policy core and secondary beliefs, as these are most relevant for understanding how obesity is framed within policymaking contexts. ‘Interests’ are defined as the ‘real, material interests of principal actors, whether conceived as individuals or as groups.’ (^(14)^, p. 176) Increasingly, these factors are recognised as part of the broader commercial determinants of health, defined as ‘the systems, practices and pathways through which commercial actors drive health and equity’^(15)^. While commercial influences have the potential to positively shape health outcomes, those associated with unhealthy commodity industries (UCI) have been regarded as key drivers of unhealthy food environments and behaviours that contribute to obesity^(15)^.

In an obesity prevention context, competing belief systems and interests are associated with differing interpretations of how obesity is defined as a problem, what causes it, who is responsible for solving it and what are the best solutions^(16)^. These dynamics are known as framing, which refers to the ways in which individuals interpret and portray a given reality by highlighting certain aspects of the problem while overlooking others that may not align with their interests^(17)^. Frames, therefore, rarely occur in a social or political vacuum. They are often a reflection of actors’ evaluation of the problem, generally informed by a combination of beliefs, interests and ideological orientations^(18)^. In an analysis of policy debates on junk-food marketing to children in Australia, Russell et al. (2020) found that parliamentarians framing of the problem aligned with their parties’ political ideology. Parliamentarians affiliated with centre-right parties framed the causes of childhood obesity in individualistic terms (e.g. poor dietary behaviours). Conversely, those from centre-left parties framed it in systemic terms (e.g. unregulated junk food marketing)^(16)^. In obesity prevention policy formulation, where contending views and trade-offs are often present^(19)^, policy actors can engage in ‘framing contests’ to shape the narrative and steer policy decisions in ways that align with their interests^(20)^. Understanding how framing dynamics occur can help identify dominant narratives that steer policy outcomes in certain directions, foster policy consensus and support evidence-informed policymaking that incorporates diverse perspectives from various stakeholders^(16)^.

Previous research investigating the contested nature of public debate associated with obesity has mostly focused on media framing^(21–23)^. Only a few studies explored the influence of frames in policy development^(16,20)^. Moreover, there remain gaps in understanding the dynamics by which the beliefs and interests of political actors inform the construction and use of frames in food and nutrition policymaking. Therefore, the aim of this study was to investigate how the different belief systems and interests of policy actors shaped their framings of the causes and solutions to obesity in their submissions to the SCOEA and how this influenced the SCOEA recommendations. The objectives of this study were to: (1) identify the actors who submitted responses to the SCOEA and their interests; (2) identify how the different actors framed obesity and examine the underlying belief systems informing their frames and (3) determine if their frames influenced the recommendations outlined in the final SCOEA report.

## Methods

### Study design

A single revelatory case study research design was adopted for this analysis. The SCOEA was chosen as the case study for two reasons. First, it provides an opportunity to identify the framings of a diverse range of actors engaged in obesity prevention policy in Australia. Second, the SCOEA report has been influential in informing the development of subsequent obesity prevention policy activity in Australia^(8)^.

### Scope and setting

#### The Select Committee into the Obesity Epidemic in Australia

Australia operates as a liberal democracy with a representative government structure comprising three levels: federal, state and territories and local governments^(24)^. The Federal Government features a bicameral parliament, which consists of two houses: the Senate and the House of Representatives. The Senate often establishes multi-partisan committees to investigate areas of public interest. These committees engage in thorough processes of investigation and deliberation, typically involving evidence collection and consultations with various stakeholders, before reporting their findings and recommendations back to Parliament^(25)^.

The SCOEA was created on 10 May 2018 by the Australian Senate to investigate obesity-related matters. Composed of five senators, one chair and one deputy chair, the Committee received 153 written submissions from individuals and organisations and conducted four public hearings between May and July 2018. In December 2018, the SCOEA released a final report with twenty-two recommendations for policymakers to tackle obesity and overweight in Australia^(11)^.

### Data collection

Submissions were extracted from the publicly accessible ParlInfo database available from the Parliament of Australia website (08/2023)^(25)^. The first author (P.R.M.) screened all documents to identify those meeting the inclusion criteria: submissions that addressed one or more enquiries. These submissions were formal written contributions provided by various stakeholders as part of the enquiry process. They aimed to address the terms of reference set out by the Committee, focusing on identifying the causes of obesity as well as proposing effective solutions. Submissions varied in content, length and detail, but generally included evidence, expert opinions and recommended strategies. Supporting documentation that did not directly address the enquiries (e.g. research papers) was excluded.

### Analytical foundations

The analysis was guided by the ‘framing matrix’ framework developed by Kwan (2009)^(26)^ and adapted by Jenkins et al. (2011)^(20)^. This framework was deemed relevant for this study due to its theoretical underpinnings rooted in framing theory and social constructivism, both of which emphasise the role of social processes and interactions in shaping individuals’ understandings of reality. Additionally, the framework was used in other studies investigating frames concerning contested public health nutrition topics^(16,20,27)^. Small adaptations were made to ensure alignment with the study’s objectives.

As illustrated in Table 1, the framing matrix framework identifies four core framing measurements: (1) position: how the problem is conceptualised; (2) causal roots: the main causes and non-causes of the problem; (3) responsibility: who is responsible for solving the problem and (4) proposed solutions.

**Table 1.** Framework used to guide the analysis

Frames	Key aspects	Prompts to identify and code data
Position	How obesity is conceptualised	- How is the issue generally described?- What is the emphasis?- What type of problem is it?
Causal roots	Main causes	- Who/what is identified as the main cause of the problem?- Who/what is to blame for the problem?
	Non-causes or less influential causes	- Who/what are dismissed or explicitly identified as non-causes?- Who/what is seen as less influential causes of the problem?
Responsibility	Responsibility for solving the problem	- Who is responsible for solving the problem?
Solutions	Proposed policy solutions	- What solutions are proposed/emphasised?

Framework adapted from Kwan (2009) and Jenkin et al. (2011).

To classify the different belief systems in obesity, a brief scoping review of public health nutrition and health promotion literature was conducted and synthesised into the framework presented in Table 2. This novel framework presents three key belief systems observed in public health nutrition policy and practice: biomedical; lifestyle and socioecological^(28–33)^.

**Table 2.** The belief systems informing the framing of the causes of and the solutions to public health nutrition problems

Criteria	Biomedical belief system	Lifestyle belief system	Socio-ecological belief system
How nutritional health is conceptualised	Nutritional health is the absence of disease. It is particularly relevant to individuals afflicted with a disease or risk factor	Nutritional health is a resource for living. It is particularly relevant to healthy individuals or individuals with risk factors for diseases	Nutritional health is a positive state of being. It is particularly relevant to healthy populations and society
Nature of associations between causes and outcomes	A linear association between a single causal risk factor and a single disease outcome (e.g. saturated fat and CVD)	A complex multifactorial association between a combination of causal risk factors and multiple health/disease outcomes (e.g. age, family history, sedentarism and poor diets are associated with non-communicable diseases)	Complex, multiple, non-linear interactive associations between disrupted food systems and multiple adverse social and ecological outcomes. (e.g. food systems and environments facilitating unhealthy foods, with minimal alternative production and distribution channels)
Causes of the nutrition problem	- Physiological predispositions to specific diseases- Nutrient imbalanced foods	- An individual’s dietary and lifestyle choices- Individual’s lack of knowledge about health	- Disrupted social and ecological settings influencing the structure and operation of food systems
Solutions to the nutrition problem	- ‘Downstream’ interventions- A focus on medical and/or technical interventions	- ‘Midstream’ Interventions- A focus on interventions that encourage individuals to change their dietary behaviours	- ‘Upstream’ interventions- A focus on addressing the social, economic, political and environmental factors influencing the structure and operations of food systems- Regulatory approaches to reduce the influence of unhealthy commodity industries
Who is responsible for solving the nutrition problem	- Health professionals, governments or specific societal sectors- Individuals are passive recipients of interventions	- Individuals are responsible for making healthier choices to improve their lifestyles- Individuals act guided by the knowledge of experts	- All sectors of society including institutions, communities, households and individuals- Governments play a key role in regulating industry practices- Individuals, particularly those experiencing high-risk conditions, have active voices in policy decisions
Examples of interventions	- Pharmaceuticals- Foods for special medical purposes- Technological innovations to reduce nutrients/ingredients to limit or increase desirable nutrients/ingredients in specific foods- Bariatric surgery	- Educational campaigns- Nutrition advice- Dietary guidelines- Voluntary food labelling or interpretative labels to inform consumer choice- Policies to nudge consumers’ behaviour through information provision rather than structural regulation	- Collaborative, whole-of-government approaches to policy- Regulatory measures targeting food industry practices- Public food procurement and food provisioning programmes- Support to producers of minimally processed foods, such as smallholder farmers- A cohesive set of policies to facilitate healthier food environments

Table developed by the authors informed by the literature^(27–32)^.

• Biomedical belief system: obesity is seen as the result of physiological dysfunctions, such as a genetic predisposition to certain diseases or a nutritional imbalance in food composition. Solutions focus on medical or technical interventions, such as bariatric surgery or reformulating specific nutrients in certain foods.

• Lifestyle belief system: obesity is attributed to poor lifestyle choices, such as dietary imbalances and sedentarism. Solutions focus on consumer education and provision of information, including educational campaigns and food labelling.

• Socio-ecological belief system: the causes of obesity encompass broader social and ecological settings that shape population health and behaviours, such as food systems that promote unhealthy food environments. Solutions focus on promoting healthier food systems and environments that facilitate the availability, accessibility and affordability of healthy foods.

### Data analysis

#### Classification of interests

An analysis of all submissions was conducted by first author (PRM) to identify and group actors who submitted enquiries based on their roles and institutional affiliation in (a) non-government organisations (NGO), (b) academia, (c) governments, (d) civil society groups and citizens and (e) the food industry and health enterprises. Their interests were classified into two primary types typically prominent in obesity prevention: commercial interests (e.g. protecting corporate profits) and non-commercial or public interests (e.g. protecting population health)^(34)^.

#### Framing analysis

Submissions were read and coded by the lead author (P.R.M.). The analysis was guided by the framework described in Table 1. The first step of analysis involved systematically identifying and coding relevant extracts of the data. A second step involved grouping initial codes into broader categories (frames) that were similar in meaning and nature. These categories were iteratively revised with co-authors to ensure they represented meaningful patterns of codes and reflected the analytical objectives of the study.

In many submissions, actors expressed multiple frames rather than adhering to a single, consistent frame. To capture the most commonly deployed frames, we categorised them as primary frames (those most prominently featured within submissions) and secondary frames (the second most prominently featured within submissions).

#### Classification of belief systems

The classification of belief systems (Table 2) was guided through iterative team discussions. Three authors (P.R.M., M.L. and T.N.) independently classified the underlying belief systems informing the frames. Inconsistencies (20 %) were resolved through a meeting until consensus (100 % consistency) was achieved.

## Results

### Identification of actors who responded to the Select Committee into Obesity Epidemic enquiries based on interests

The SCOEA received 153 written submissions from individuals and representatives of organisations or institutions^(11)^. Two submissions were confidential and unavailable for access. One submission was not assessed due to submitter’s name being withheld. Table 3 shows the actors who submitted their responses to the enquiries and their interests. Of the 150 available submissions, 120 submitters were identified as groups or individuals with non-commercial interests. These included governments and government-related institutions (*n* 13), NGO (*n* 49), civil society groups and citizens (*n* 24) and academics (*n* 34). A total of thirty submitters were determined to have commercial interests in the topic at hand. These included representatives of the food industry and industry bodies (*n* 23) and health enterprises (*n* 7). A list containing all submitters can be found in Table S1 (see online supplementary material, Supplemental material).

**Table 3.** Identification of the actors who responded to the Select Committee on Obesity Epidemic in Australia enquiries and classification of their potential interests

Groups and individuals	*n*
Actors associated with non-commercial interests	
Governments and government-related institutions	13
Non-government organisations (independent of government and industry groups) Examples: non-government organisations, advocacy groups, associations, trade unions or societies	49
Civil society groups and citizens	24
Academia Examples: members from universities/research institutes, expert groups, Think-Tanks	34
Total	120
Actors associated with commercial interests	
Food industry and related industry bodies Description: organisations engaged in food-related commercial, industrial or professional activities. Industry bodies are considered organisations founded and funded by businesses that operate in a specific industry such as a trade association, industry trade group or business association	23
Health enterprises description: organisations engaged in commercial activities related to health promotion, e.g. medical, or pharmaceutical companies or wellness enterprises	7
Total	30
Total submissions analysed	150

### Framing and belief systems

Structured around four framing components – *position, causal roots, responsibility and solutions* – this section presents the results of the framing analysis. Table 4 displays the primary and secondary frames deployed by the actor groups and their underlying belief systems.

**Table 4.** Identification of how different policy actors framed the causes of and the solutions to the rise of overweight and obesity and which types of belief systems were represented in their frames

Actor	Primary frame^*^	Secondary frame^*^
Framing dimension 1 – Position		
Non-government organisations	Obesity is a multifactorial, complex problem, influenced by a range of individual and environmental-level factors, such as cultural and socio-economic contexts. Belief system: socioecological	Obesity is a chronic disease, or a metabolic condition, characterised by a dysregulation of energy stores and an imbalance of important hormonal and neural pathways. Belief system: biomedical
Academia	Obesity is a complex and multifactorial problem influenced by a range of broader interrelated factors (e.g. environmental, socioeconomic and cultural factors) that can shape individual’s health and behaviours. Belief system: socioecological	Obesity is a metabolic condition influenced by two main pathways (i) a developmental pathway that starts in the womb and which affects biological mechanisms of obesity risk and (ii) an environmental pathway that triggers dormant genetic processes and influences risk behaviours. Belief system: biomedical
Governments	Obesity is a multifaceted problem of energy imbalance influenced by a range of inter-related factors, both internal and external to the individual, that have different impacts among populations. Belief system: socioecological	Not identified
Civil society groups and citizens	Obesity is a problem of energy imbalance caused by high energy intake and low energy expenditure. Belief system: lifestyle	Obesity is a social problem, influenced by historical and social factors that have shaped eating patterns and physical activity levels in modern societies. Belief system: socioecological
Food industry, industry bodies and health enterprises	The food industry and associated bodies framed obesity as a complex and multifactorial problem, whereby complex interactions between genetic, behavioural, cultural, environmental and socio-economic factors affect energy balance. There is no single cause, nor a single solution. Belief system: socioecological	Health enterprises framed obesity as a complex chronic disorder resulting from poor metabolic health. Belief system: biomedical
Framing dimension 2 – Causal roots		
2·1 Main causes		
Non-government organisations	Environmental causes, particularly the obesogenic food and built environments that facilitates access to unhealthy foods and drinks while hindering individuals’ ability to engage in physical activities that promote health and well-being. Belief system: socioecological	Modern lifestyles are characterised by poor dietary behaviours and insufficient levels of physical activity and/or sedentarism. Belief system: lifestyle
Academia	Socio-economic factors such as urbanisation and industrialisation, have created food environments that facilitate the consumption of nutritional poor and energy-dense foods while hindering individuals’ ability to engage in physical activities that promote health and well-being. Belief system: socioecological	Biological mechanisms, such as genetic variations and epigenetic programming, can influence an individuals’ appetite and responses to food. Parental body mass is also an important influence on the weight of future generations. Belief system: biomedical
Governments	The broader environments in which people participate and live shape health behaviours and health status. Key environmental factors that contribute to obesity are (i) nutrient content of meals, foods and drinks purchased outside of the home; (ii) unhealthy food and marketing and advertising; (iii) the built environment and (iv) unhealthy school environments for children. Belief system: socioecological	A myriad of behavioural risk factors influences weight gain and health status. These include poor compliance with guidelines for healthy eating, parental modelling influencing children’s food choices and fragmented family structures impacting eating habits. Belief system: lifestyle
Civil society groups and citizens	The food industry is responsible for perpetuating obesity by (i) manufacturing unhealthy food products; (ii) implementing poorly regulated marketing and advertisement mechanisms; (iii) promotion of unhealthy food products via a flawed front-of-pack labelling system and (iv) posing resistance to stricter government regulation of foods. Belief system: socioecological	Changes in food consumption patterns, physical inactivity and sedentarism lead to energy imbalance. These changes have resulted from the latest modern life events, such as women entering the workforce, longer working hours and less active travel. Belief system: lifestyle
Food industry, industry bodies and health enterprises	Individuals not complying with guidelines for healthy eating or not meeting the Five Food Group recommendations. Belief system: lifestyle	Health enterprises identified individual-level factors such as working patterns, diets and energy expenditure and genetic, as major contributors to weight gain. Belief system: lifestyle
2·2 Non-causes or secondary causes		
Non-government organisations	The notion of individual responsibility must be placed in the context of the capacity that individuals have to respond to the obesogenic environment they participate and live. Belief system that is not being supported: lifestyle	Not identified
Academia	Individuals have the least influence over their food choices when compared to the broader external forces that shape dietary behaviours. Belief system that is not being supported: lifestyle	Not identified
Governments	Obesity is not simply a matter of personal choice, but a social condition for which governments and relevant stakeholders have a responsibility to solve. Belief system that is not being supported: lifestyle	Not identified
Civil society groups and citizens	Not identified	Not identified
Food industry, industry bodies and health enterprises	Obesity is multifactorial and therefore, a single nutrient or a single food, is not their main driver. Insufficient information was provided to identify the underlying belief system	Advertising is one of the many drivers of obesity, not the main one. Evidence of its association with obesity is weak and inconclusive. Insufficient information was provided to identify the underlying belief system
Framing dimension 3 – Responsibility		
Non-government organisations	All levels of government and relevant stakeholders, including representatives of the private sector. Belief system: socioecological	While the food sector has a role to play in helping attenuate obesity, their participation in policy decisions must be limited to the latest stages of policymaking. Insufficient information was provided to identify the underlying belief system
Academia	A societal response that includes all levels of governments, relevant stakeholders and a wide-scale action from the food industry. Belief system: socioecological	While the food sector has a role to play in helping attenuate obesity, their participation in policy decisions must be limited to the latest stages of policymaking. Insufficient information provided to identify the underlying belief system
Governments	All levels of governments and societal sectors including, non-government organisations, consumer groups, the private sector and individuals to coordinate a collaborative approach to address obesity. Belief system: socioecological	The food sector has a role to play in helping attenuate obesity in Australia. Insufficient information was provided to identify the underlying belief system
Civil society groups and citizens	Governments are responsible to attenuate obesity through the implementation and monitoring of strict regulatory activities. socioecological	While the food sector has a role to play in helping attenuate obesity, their participation in policy decisions must be limited to the latest stages of policymaking. Insufficient information was provided to identify underlying belief system
Food industry, industry bodies and health enterprises	Resolving the problem of obesity is the responsibility of governments, the food sectors and other relevant stakeholders. Belief system: socioecological	The food industry can help people make healthier food choices through a range of voluntary self-initiated actions and compliance with existing policies. Insufficient information provided to identify underlying belief system
Framing dimension 4 – Solutions		
4·1 Proposed solutions		
Non-government organisations	Creating supportive environments to help people make healthier choices through (i) placing a health levy on sugar-sweetened beverages; (ii) government-led regulations on the marketing of unhealthy foods to children; (iii) improving the Health Star Rating system; (iv) setting clear reformulation targets for food manufacturers; (vi) efficient urban planning and (v) improvements to school food environments. Belief system: socioecological	Providing guidance and support to encourage healthy eating and increase physical activity. Belief system: lifestyle
Academia	Creating supportive environments to help people make healthier choices through: (i) improving the Health Star Rating system; (ii) government regulation on the food industry with focus on the marketing of foods to children; (iii) taxation of unhealthy foods and drinks; (vi) food reformulation to improve the nutritional profile of food products and (v) improvements to school food environments. Belief system: socioecological	Establishing obesity prevention as a national priority with sustained funding, regular monitoring, evaluation of key measures and reporting around targets. This comprehensive approach to obesity should be underpinned by a National Obesity Strategy and coordinated by a National Obesity Taskforce. Belief system: socioecological
Governments	High impact sustained public education campaigns to improve dietary behaviours, physical activity and sedentary behaviour. Belief system: lifestyle	A combination of interventions spanning a set of priority areas of (i) laws, regulations and taxes to reduce the availability of unhealthy foods and facilitate the access to healthy foods; (ii) promotion of healthy food environments within school settings; (iii) clinical service delivery and community-based approaches to treat those affected by obesity and (vi) policies to facilitate physical activity and promote active travel. Belief system: socioecological
Civil society groups and citizens	Time-based legislative restrictions on children exposure to unhealthy food and drink marketing on free-to-air television and banning of advertising of unhealthy foods in sport events Belief system: socioecological	Placing a health levy on sugary drinks to increase the price by 20 %. Belief system: socioecological
Food industry, industry bodies and health enterprises	Used terms as ‘whole-of-system’, ‘system-thinking’ and ‘collaborative’ to propose education strategies to educate consumers on healthy eating and physical activity and/or multistakeholderism. Belief system: lifestyle	Not identified

* The descriptions of both primary and secondary frames reflect the general state of the framing dimensions depicted by the submitters across the different actor groups.

### Position (how actors conceptualised obesity)

Frames conceptualising obesity were typically presented in the introductions of submissions, setting the tone for subsequent arguments and recommendations.

In line with the socio-ecological belief system described in Table 2, the majority of actors from most groups (except for civil society submitters), primarily conceptualised obesity as a complex and multifactorial condition influenced by a range of interrelated factors, both internal and external to the individual. While they recognised the sociological drivers of obesity, their framings differed in emphasis. NGO focused on the roles of cultural and socio-economic contexts as key contributors to obesity. Academics emphasised how these factors shape individual health and behaviour, whereas governments framed them in terms of their impacts on energy imbalance and how these can differ according to population. Industry actors highlighted the complexity of the problem emphasising that there is no single cause or solution to obesity.

Civil society groups, however, diverged from this multifactorial framing. Instead, most submitters from this group primarily conceptualised obesity in lifestyle terms, framing it as a chronic condition resulting from poor lifestyle choices, such as unhealthy eating and physical inactivity.

Other commonly identified frames among several NGO, health enterprises and academics were those informed by a biomedical belief system, under which obesity was conceptualised as a metabolic condition caused by physiological body disruptions. When deployed by academics, this frame was often complemented with an encompassing understanding of nutritional health that acknowledged the obesogenic environment as an important contributor to weight gain.

### Causal roots

#### Main causes

Causation frames were typically deployed by submitters when responding to the enquiry on the causes of the rise in overweight and obesity in Australia.

Aligned with a socio-ecological belief system, most actors across all non-commercial groups emphasised the broader structural conditions that influenced weight gain when framing obesity causation. In their submissions, a significant focus was given to the ‘obesogenic environment’ in which individuals participate and live. The term ‘obesogenic’ was used by several submitters, generally when referring to environments *‘conducive to unhealthy behaviours and where unhealthy food and poor physical activity choices are the easy and normal choices.’* However, their emphases on specific environmental factors varied. Academics highlighted broader drivers such as urbanisation and industrialisation. Governments identified key environmental contributors, such as the nutrient content of meals, unhealthy marketing and the built environment. Civil society and citizen groups were more specific in holding the food industry accountable for perpetuating obesity. For example, one submission from a citizen highlighted the food industry’s resistance to regulatory interventions and its role in promoting sugar in the food supply:

*‘It [the food industry] has resisted appropriate regulation (front of pack labelling) so that consumers do not discriminate based on sugar content. It is largely responsible for ensuring that sugar is embedded throughout our food supply.’*

A lifestyle-informed frame of obesity causation was prevalent across most actor groups, particularly commercial submitters, for whom it emerged as the primary frame. Most industry submitters placed the blame onto individuals for not complying with dietary guidelines. Health enterprise submitters identified a myriad of individual-level factors, such as lifestyle and genetic predisposition. Among non-commercial submitters (e.g. NGO and governments), lifestyle narratives emerged as a secondary frame. In their submissions, most actors emphasised a range of behavioural risk factors, encompassing both food-related causes (e.g. poor adherence to healthy eating guidelines) and non-food-related factors (e.g. physical inactivity and sedentarism).

#### Non-causes

Contesting a lifestyle-informed frame, non-commercial actors did not view individual behaviour as the main cause of obesity. Instead, most of them viewed obesity as a consequence of a poorly regulated environment that facilitated unhealthy lifestyles. A submission from an NGO focused on health and wellbeing illustrates this point:

*‘What combination of factors cause this [obesity] is not completely known, but the causes are certain to lie in the food environment, and the physical activity environment.’*

Most commercial actors agreed on the multifactorial nature of obesity. Several argued against attributing its rise to a single nutrient (e.g. sugar) or food (e.g. sugary drinks). This position was often supported by references to data showing a negative correlation between sugar consumption and obesity rates over time. A submission from a multinational beverage corporation exemplified this point:

*‘US obesity rates have more than doubled between 1990 and 2015, while sugar intake fell. On the facts, it seems reasonable to conclude that neither sugar, nor drinks sweetened with sugar, were the major cause of this obesity rise.’*

Using similar logic, all industry actors involved in advertising activities argued that the marketing of unhealthy foods was not the main cause of obesity and that evidence of its association with weight gain was inconclusive.

### Responsibility (who is responsible for solving the problem)

Frames surrounding responsibility for solving obesity were generally deployed by submitters when responding to the enquiry on evidence-based measures to prevent and reverse childhood obesity.

The majority of submitters from most groups (except for civil society and citizens) primarily framed obesity as a societal problem that required a collaborative approach for effective intervention; a way of framing consistent with a sociological belief system. Several actors across all groups acknowledged the food industry’s role in providing healthier foods in the supply chain, particularly through improvements to the nutritional profile of their products. However, non-commercial actors expressed reservations about the extent to which the industry sector should be involved in policy formulation. Due to their irreconcilable conflict of interests, several submitters across different groups (i.e. NGO, academics and civil society groups) believed that the food industry’s participation in policy should be limited to the final stages of decision-making. This is illustrated in the below quote from a citizen:

*‘In seeking solutions to obesity, it will be important to engage experts who can take an evidence-based approach. The food industry will need to be involved, but only after the parameters have been set by those without any conflict of interest.’*

Most civil society and citizen submitters emphasised the role of governments in addressing obesity through the implementation and monitoring of strict regulatory measures.

Industry actors emphasised their efforts to address obesity, outlining their responsibilities in detail. These included self-initiated measures such as labelling information, improving product portfolios, portion resizing and compliance with existing policies and regulations.

### Solutions

#### Proposed policy solutions

Frames proposing solutions to obesity were generally deployed by submitters when responding to the enquiry on evidence-based measures to prevent and reverse childhood obesity.

In accordance with a socio-ecological belief system, several actors from NGO and academia groups primarily framed obesity as a complex problem requiring a comprehensive policy approach spanning multiple areas. Similar perspective was deployed by governments as a secondary frame. Broadly, submitters proposed a combination of interventions aimed at creating supportive environments that promote healthier behaviours. Commonly identified solutions were:

(i)Placing a 20 % health levy (flat-rate energy tax) on sugar-sweetened beverages.

(ii)Enforcing time-based restrictions on the marketing of unhealthy foods and drinks to children, with a particular focus on free-to-air television platforms.

(iii)Mandating the front-of-pack labelling system, the Health Star Rating, and reviewing its algorithm.

(iv)Setting clear reformulation targets for food manufacturers with specific timelines to ensure compliance.

(v)Improving school food environments.

(vi)Provision of clinical treatment and community-based approaches to treat those affected by obesity.

(vii)Improvements to the structure of built environments and adequate urban planning to facilitate active travel.

Consistent with a socio-ecological belief system, several academics framed obesity as a ‘national priority’ (secondary frame) and proposed a comprehensive and coordinated approach to address it, with the establishment of a National Obesity Strategy and task force. Civil society and citizens advocated for legislative measures to nudge consumers towards healthier food choices (e.g. marketing restrictions on unhealthy foods), indicating a preference for regulatory approaches to obesity.

Lifestyle-informed solutions to address obesity emerged as a primary frame among both government and industry groups. Most submitters from these groups emphasised education campaigns to change individual behaviour as primary solutions. Industry submitters, in particular, used terms such as ‘whole-of-system’, ‘system-thinking’ and ‘collaborative’ when framing potential solutions. However, these frames did not extend to system-level recommendations and remained focused on individual-level interventions. Additionally, industry submitters emphasised the need for a multistakeholder approaches, highlighting collaboration between the public and private sectors. A national industry association representing food and grocery manufacturers illustrated this view:

*‘A well-considered, evidence-based approach where all players have a role in supporting a consistent and united strategy, based not only on products, but also diets, education and activity, is the way forward. […]the Australian food industry is ready and well-positioned to be a key contributor.’*

### Identification of the underlying belief systems represented in the Select Committee into Obesity Epidemic final report

**Figure 1.**
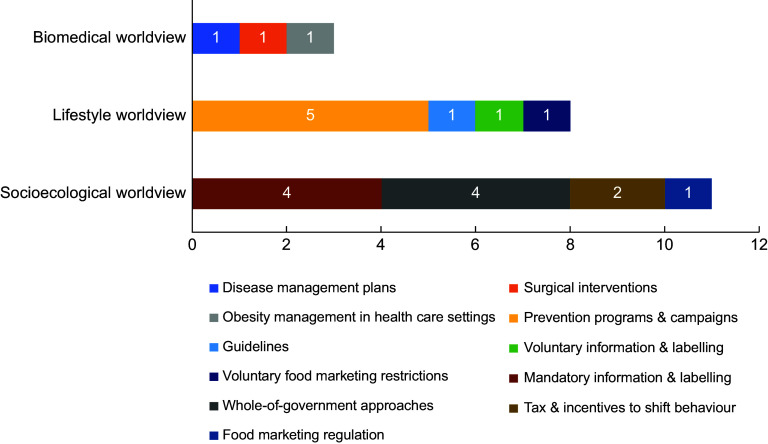
Representation of the underlying belief systems informing the policy recommendations to halve the rise of obesity in Australia outlined in the SCOEA 2018 final report.

Figure 1 shows the representation of underlying belief systems informing the SCOEA report’s recommendations. Of the twenty-two recommendations to address obesity, eleven were sociologically informed. These included comprehensive and multi-sectoral approaches through the establishment of a National Obesity Strategy and task force (*n* 4), mandatory information and labelling (*n* 4), tax and incentives to shift behaviour (*n* 2) and food marketing regulation (*n* 1). Lifestyle-informed recommendations (*n* 8) included educational campaigns and preventive actions (*n* 5), reviewing dietary guidelines (*n* 1), voluntary information and labelling (*n* 1) and voluntary food marketing restrictions (*n* 1). Biomedical recommendations (*n* 3) included strengthening obesity care in medical settings (*n* 1), educating medical professionals on bariatric procedures (*n* 1) and adding obesity to the Chronic Disease Management scheme (*n* 1). Detailed information about the recommendations can be found in Table 5.

**Table 5.** Representation of the underlying belief systems informing the policy recommendations to halve the rise of obesity in Australia outlined in the SCOEA 2018 final report

Biomedical recommendations	Lifestyle recommendations	Socio-ecological recommendations
1. The Medical Services Advisory Committee to consider adding obesity to the list of medical conditions eligible for the Chronic Disease Management scheme	1. Overweight and obesity prevention and treatment programmes to be contingent on the appropriate use of language to avoid stigma in public health campaigns, programme design and delivery	1. The establishment of a National Obesity Taskforce, comprising representatives across all knowledge sectors from federal, state and local government and alongside stakeholders from the NGO, private sectors and community members. The Taskforce is to be responsible for all aspects of policy direction, implementation and the management of funding
2. The Australian Medical Association, the Royal Australian College of General Practitioners and other college of professional bodies to educate their members about the benefits of bariatric surgical interventions for some patients	2. The Australian Dietary Guidelines are to be updated every 5 years	2. The establishment of a National Obesity Strategy in consultation with all key stakeholders across government, the NGO and private sectors
3. The Commonwealth Department of Health work with organisations responsible for training medical and allied health professionals to incorporate modules aimed at increasing awareness of stigma and blame in medical, psychological and public health interventions for overweight and obesity	3. Reviewing of voluntary front-of-pack labelling schemes to ensure they are fit-for-purpose and adequately represent the nutritional value of foods and beverages	3. The National Obesity Taskforce to form a sub-committee directly responsible for the development and management of a National Childhood Obesity Strategy
	4. Free TV Australia to voluntary introduce restrictions on discretionary food and drink advertising on free-to-air television until 21.00	4. The National Obesity Taskforce to develop a National Physical Activity Strategy
	5. The National Obesity Taskforce is funded to develop and oversee the implementation of a range of National Education Campaigns with different sectors of the Australian community	5. Improvements to the Health Star Rating system, including (a) its calculator to address inconsistencies; (b) industry sectors to be removed from the Advisory Group and (c) the Health Star Rating system to be made mandatory by 2020
	6. The National Obesity Taskforce commission evaluations are to be informed by multiple methods of past and current multi-strategy prevention programmes with the view of designing future programmes	6. The adoption of mandatory labelling of added sugar on packaged foods and drinks
	7. The National Obesity Taskforce is funded to develop and oversee the implementation of multi-strategy, community-based prevention programmes in partnership with communities	7. Development of a consistent and mandatory nutritional information label for fast food menus across all government jurisdictions
	8. The National Obesity Taskforce is funded to develop and oversee culturally appropriate prevention and intervention programmes for Aboriginal and Torres Strait Islander communities	8. The Health Star Rating is to be mandatorily displayed on food and beverage products advertised on all forms of media
		9. Introduction of a tax on sugar-sweetened beverages, with the objectives of reducing consumption, improving public health and accelerating the reformulation of products
		10. The Australian Government to consider introducing legislation to restrict discretionary food and drink advertising on free-to-air television until 21.00 if these restrictions are not voluntarily introduced by Free TV Australia by 2020
		11. The development of initiatives and incentives to increase access, affordability and consumption of fresh foods in remote Aboriginal and Torres Strait Islander communities

## Discussion

In this study, we aimed to investigate how the different belief systems and interests of a diverse range of actors influenced obesity framing and how these underlying dynamics were reflected in the final SCOEA report. Broadly, five key findings emerged from our analysis.

First, there were notable similarities in how commercial and non-commercial actors conceptualised obesity. Both groups portrayed obesity as a complex condition influenced by a range of external factors beyond individual control; a way of thinking consistent with a socio-ecological belief system. Increasingly, the notion of obesity as a complex issue has been propagated among public health groups, including governments^(16)^, public health advocates and other expert professionals^(35)^. However, the use of complex language has also gained traction within the industry sector, likely as a strategy to dispute their roles in contributing to the problem and to delay effective action, notably those involving stricter regulation^(36)^.

Second, significant differences between how commercial and non-commercial actors framed the causes and solutions to obesity were observed. Consistent with previous findings^(20)^, non-commercial actors predominantly framed obesity in socio-ecological terms, viewing weight gain as a natural response to an obesogenic environment that promotes unhealthy behaviours while contesting the lifestyle frame of individual responsibility. In contrast, commercial actors framed obesity as a lifestyle issue, attributing it to poor individual behaviour. This framing strategy, commonly referred to as ‘blame-shifting,’ is a well-documented industry tactic whereby commercial actors shift the responsibility away from themselves and onto other actors, particularly consumers so that attention is focused on personal behaviours rather than systemic industry-driven factors^(37)^. Additionally, these actors rejected the idea of *‘*single causality’, emphasising that obesity is a complex and multifactorial problem and, therefore, no single nutrient (e.g. sugar), food (e.g. sugary drinks) or factor (e.g. marketing) should be viewed as its main driver.

Regarding solutions, non-commercial submitters frequently proposed both socio-ecological and lifestyle approaches to obesity. Socio-ecological frames emphasised a cohesive set of interventions to create supportive environments conducive to healthy behaviours and regulatory measures to industry practices, such as marketing restrictions on unhealthy foods and a healthy levy on sugary drinks. The use of sociologically informed frames by non-commercial actors has been documented in previous framing analyses^(16,38)^, reflecting these actors’ focus on addressing the broader determinants of health. Lifestyle frames mostly emphasised prevention programmes and educational campaigns. Contrastingly, industry actors exclusively supported lifestyle interventions centred on consumer education and voluntary industry measures. Additionally, they frequently proposed collaborative approaches involving the private sector, likely to maintain influence in decision-making and ensure that policies align with their interests. Despite using system-level language such as ‘whole-of-system’ and ‘system-thinking’, they refrained from engaging with system-change solutions to solve what they had initially conceptualised as a complex problem. This strategy, described by Campbell et al. (2020) as ‘co-opting’, involves assimilating elements from opposing groups to gain legitimacy and deflect regulatory actions targeting their products or practices^(37)^.

Third, while non-food-related frames associated with physical activity and clinical treatments for obesity emerged, this analysis revealed a dominant focus on food and diet-related narratives. The emphasis on foods and diets in obesity prevention discourse is likely a reflection of the central roles these factors play in weight gain and poor health (6·7) Additionally, the profile of submitters may have contributed to this skewing. A significant proportion of submissions were from stakeholders or organisations associated with food and/or nutrition activities, with relatively few representing perspectives on other determinants of obesity. It is notable that, despite the increasing emphasis on the broad socio-ecological drivers of obesity in the literature^(4)^, factors such as inequality, deprivation or marginalisation were not mentioned in the submissions. This omission has been previously reported in the literature^(38)^ and might indicate challenges in integrating equity into mainstream public health narratives.

Fourth, while policy actors’ interests significantly shaped their frames (especially commercial submitters, who employed strategic framing to protect their interests), actors’ underlying belief systems also played a substantial role. This was particularly evident among non-commercial actors, where a broader diversity of frames emerged. For example, civil society groups primarily conceptualised obesity as an energy imbalance issue, while the other actor groups emphasised its multifactorial nature. Academics often adopted a biomedical perspective, emphasising genetic and epigenetic factors as key contributors to obesity, whereas the remaining groups focused on lifestyle factors like unhealthy diets and reduced physical activity. Regarding solutions, NGO and academics generally advocated for socio-ecological approaches that involved creating supportive environments conducive of healthier behaviours, while governments largely promoted lifestyle interventions, such as educational campaigns. This diversity of views, even among actors within the same group, is not an uncommon observation in public health nutrition, reflecting its complexity, interdisciplinary foundations and multiplicity of stakeholders^(39,40)^. While this plurality may facilitate a more nuanced understanding of public health problems, it can also pose challenges to consensus building and implementation of effective policies^(41)^.

Lastly, the policy recommendations made by the Committee on how to halve obesity and overweight in Australia encompassed a broad range of solutions endorsed by various submitters. Most of these recommendations consisted of sociological and lifestyle interventions, broadly aligning with those proposed by both commercial and non-commercial groups. Importantly, the influence of commercial actors was notable in the Committee’s reluctance to adopt stricter regulatory measures. Despite significant calls from non-commercial actors for a regulatory approach to food advertising, the Committee deferred responsibility to implement voluntary restrictions to the Free TV Australia, an industry body representing television broadcasters^(11)^. This ability of commercial actors to influence both discourse and policy outcomes reflects the extensive power of commercial determinants of health in policymaking processes, aligning with previous research on the influence of UCI, such as tobacco, alcohol and the food industry, on policy decision-making^(42–44)^.

The findings from this study are significant, as they illustrate how belief systems and interests of policy actors can shape their framing of an important public health nutrition problem and, ultimately, influence the development of a key document used to inform obesity prevention policy. This study demonstrates that policy decisions rarely occur in a social and political vacuum. More often than not, they are largely a reflection of the beliefs and interests of policy actors, typically those with the greatest power^(45)^. While an inherent aspect of policymaking, these hidden dynamics can often steer policy outcomes in undesirable directions. In Australia’s obesity prevention context, for example, the dominance of a belief system rooted in individual responsibility, combined with the pervasive influence of corporate interests on policy decisions, has resulted in a significant inaction in critical areas, most notably those that align with a socio-ecological thinking, such as marketing regulation to children, mandatory front-of-pack labelling and fiscal policies^(3,46)^.

To address these challenges and promote greater transparency and inclusivity in consultation processes, four recommendations are proposed. First, active engagement with a diverse range of stakeholders (particularly those most affected by the harmful practices of UCI) is needed to design effective solutions^(47)^. Second, submissions should be more critically evaluated by decision-makers to identify strategic framing approaches and counteract industry narratives through tools like framing analysis^(47)^. Third, the influence of UCI on consultation settings must be mitigated through stronger governance mechanisms, such as public disclosure and reporting of funding sources^(48)^. Finally, to effectively tackle the obesity crisis, policy decisions themselves need to be more ambitious and systemic in scope. While lifestyle solutions have their place, they must be complemented by a cohesive set of policies aimed at addressing the social, commercial and political determinants of health^(49)^.

### Strengths and limitations

This study has several strengths. First, to the best of the author’s knowledge, it is the first to investigate the role of belief systems and interests in obesity framing among a diverse range of actors involved in food and nutrition policymaking. Second, the results were strengthened by using a validated framework previously utilised by other authors investigating contested public health nutrition issues^(16,20,27)^. Third, the study’s social constructionist approach and focus on belief systems and interests address an underexplored area in public health nutrition and make a significant contribution to advancing new knowledge.

This study also has some limitations. First, the overlapping characteristics of different belief systems can lead to difficulties in frame classification, as a frame (or the interventions proposed under it) may span both lifestyle and socio-ecological perspectives. For example, while regulatory measures like mandatory labelling can influence individual behaviour (lifestyle), their primary focus is on regulating industry practices, and they were therefore classified under a socio-ecological belief system. To address this, inter-rater reliability checks were conducted to ensure robust classification. Second, actors’ interests may not always be clear. Consequently, underlying interests that could shape their frames (e.g. political gains or professional advancement) might not have been fully captured in this analysis. Future research should consider a more nuanced investigation of a diverse range of actors’ interests to provide a comprehensive understanding of how these can influence policy.

Lastly, the ratio between commercial (*n* 30) and non-commercial submissions (*n* 120) may appear skewed, potentially raising concerns about bias. However, this likely reflects the natural presence and distribution of policy actors in health policymaking settings, where non-commercial actors such as expert professionals and civil society actors are higher in number. However, this study shows that despite their smaller numbers, commercial actors had significant influence over policy decisions.

### Conclusion

Competing belief systems in the framing of obesity were identified among submitters, particularly between commercial and non-commercial groups. Non-commercial actors framed obesity in biomedical, lifestyle and socio-ecological terms, emphasising genetics, individual agency and the obesogenic environment’s role in weight gain. Their proposed solutions encompassed lifestyle measures and socio-ecological approaches.

In contrast, commercial actors used complex language to conceptualise obesity but did not engage with system change solutions. Instead, they framed the issue as one of individual behaviour and exclusively supported consumer education interventions. These conflicting frames reflect a key tension in obesity prevention efforts: while non-commercial actors advocate for substantial reforms and, in some cases, transformative policies, commercial actors favour incremental adjustments that shift responsibility to individuals and require minimal changes to their practices. Governments may find commercial frames appealing, as their proposed solutions are less disruptive and easier to implement. However, individual-level approaches focused on consumer education, while valuable, fail to address the structural drivers of obesity and do little to promote transformative change.

This study shows that policy actors’ framing is shaped by their belief systems and interests, ultimately influencing the development of a key policy report in Australia. Understanding the social and political variables that inform food and nutrition policy, including the overlap between belief systems and strategic interests, is critical. While genuine beliefs might guide ethical action, strategic interests, especially those from UCI, must be approached with caution. Policymakers seeking to advance effective obesity prevention policy must critically evaluate the framing strategies used by various actors with competing interests ensuring that policy decisions are grounded in relevant evidence and aligned with health, equity and ecological perspectives.

## Supporting information

Ribeiro de Melo et al. supplementary material Ribeiro de Melo et al. supplementary material

## Supplementary material

For supplementary material accompanying this paper visit https://doi.org/10.1017/S1368980025100517
