# Supplementary material for: The role of policy actors’ belief systems and interests in framing public health nutrition problems: a case study of obesity in Australia
Source: Public Health Nutr. 2025 Jun 3;28(1):e103. doi: 10.1017/S1368980025100517 (PMC12264778; doi:10.1017/S1368980025100517)
Supplement: Ribeiro de Melo et al. supplementary material [file S1368980025100517sup001.docx]

**Table S1** List of submitters who responded to SCOEA 2018 enquiries

| **Governments and government-related institutions** | **Non-government organisations** | **Civil society groups and members from the general community** | **Academia** | **Food industry** | **Industry bodies** | **Health enterprises** |
| --- | --- | --- | --- | --- | --- | --- |
| 1. Nepean Blue Mountains Family Obesity Service  2. Australian Institute of Health and Welfare  3. Health Star Rating Advisory Committee (HSRAC)  4. ACT Government  5. **Live Lighter WA**  **6. City of Greater Bendigo**  **7. Government of Western Australia**  **8.** Australian Local Government Association  **9. Northern Territory Government**  **10. City of Cockburn**  **11. Australian Government Department of Health**  **12. Tasmanian Government**  **13. NSW Health** | 1. Early Life Nutrition Coalition  2. Council of Presidents of Medical Colleges  3. YMCA Victoria  4. The Victorian Centre of Excellence in Eating Disorders (CEED)  5. Eating Disorders Victoria  6. Lactation Consultants of Australia and New Zealand  **7. Food Fairness Illawarra**  8. Primary Care Partnership  9. Royal Australian and New Zealand College of Psychiatrists  **10. Sugar Free Smiles**  11. Cancer Council Australia  12. Sugar By Half  13. Queensland Nurses and Midwives’ Union (QNMU)  14. Queensland Country Women's Association  15. Australian Health Policy Collaboration  16. Filter Your Future  17. **Nutrition Australia**  **18. Diabetes Australia**  **19.** Catholic Women’s League Australia  20. The Obesity Collective  **21. Australian Sugar Alliance**  22. Grains & Legumes Nutrition Council  **23. Public Health Association of Australia**  **24. The Australian Prevention Partnership Centre**  25. Exercise and Sports Science Australia (ESSA)  26. World Breastfeeding Trends Initiative  27. NCDFREE  28. Partners in Prevention Geelong (Dr Nicholas Brayshaw)  29. Partners in Prevention Geelong  30. Choice  31. Australian and New Zealand Metabolic and Obesity Surgery Society  32. South Australian Nutrition Network (SANN)  33. Butterfly Foundation  34. Australian Healthcare and Hospitals Association  35. Breastfeeding Coalition Tasmania  36. Australian Chronic Disease Prevention Alliance  37. Dietitians Association of Australia  38. Australian Council of Social Service  39. That Sugar Movement  40. Australian Taxpayers’ Alliance  41. **Australian Medical Association**  **42. Consumers Health Forum of Australia**  **43.** Services for Australian Rural and Remote Allied Health  44. **National Aboriginal Community Controlled Health Organisation**  45. Gold Coast Health and Wellbeing Working Group  **46. National Rural Health Alliance**  47. Heart Foundation  48. Cockburn Integrated Health  49. Arthritis Australia | 1. Lance Payne  **2. David Roberts**  3. David Hale  4. Amanda Atkins  5. Leanne Chambour  6. Alan Barclay  7. Ingrid Ozols  **8. David Gillespie**  9. Parents’ Voice  **10. Marcea Klein**  **11. Federation of Parents and Citizens Associations of NSW**  12. Edward Cliff  13. Terry Barnes  14. Tom Scully  15. Caroline Miller and Aimee Brownbill  16. Rahul Barmanray  17. Leonie Elizabeth  18. Rosemary Stanton  19. Jim Donovan  20. Rory Robertson  21. Gary Fettke  22. Luigi Fontana  **23. Melinda Stratton**  **24.** Greg Stewart | 1. Jennifer Thompson  **2. Centre for Research Excellence in Integrated Quality Improvement**  3. WA Cancer Prevention Research Unit  4. Centre for Research Excellence in the Early Prevention of Obesity in Childhood  5. Jonathan James Pincus, FASSA  6. Global Obesity Centre (GLOBE)  7. Monash Centre for Health Research and Implementation  8. The Royal Children’s Hospital Melbourne  9. Australian College of Nursing  10. International Health Economics Association (iHEA), Economics of Obesity Special Interest Group (EOSIG)  **11. National Centre for Epidemiology and Population Health, The Research School of Population Health, at The Australian National University**  12. The Baker Heart and Diabetes Institute  13. Flinders University  14. Dr Narelle Story - Faculty of Medicine and Health  15. The Children’s Hospital at Westmead  16. Institute for Physical Activity and Nutrition at Deakin University  17. Grattan Institute  18. School of Social Sciences, The University of Adelaide  19. Queensland Child and Youth Clinical Network  20. Priority Research Centre for Physical Activity and Nutrition, Nutrition and Dietetics  21. Food Governance Node  22. Dr Shannon Sahlqvist and Alfred Deakin Professor Anna Timperio  23. Food and Movement Research Team at Early Start, University of Wollongong  24. Swinburne University of Technology  25. Menzies School of Health Research 26. Mark Lawrence, Institute for Physical Activity and Nutrition and School of Exercise and Nutrition Sciences, Deakin University  **27. Menzies Institute for Medical Research**  **28. The George Institute**  29. Professor Wendy A. Brown – Monash  30. The National Health and Medical Research Council  31. Menzies Research Centre  32. The Boden Institute University of Sydney  33. Obesity Policy Coalition (Think Tank)  34. Joep Lange Institute | 1. Robert Lowndes (New Zealand Sugar Company Ltd)  3. Kentucky Fried Chicken  4. Fonterra Australia  5. Nestle Australia  6. Robern Menz (Mfg) Pty Ltd  7. Coca-Cola Amatil  8. Haigh's Chocolate  9. Mars Australia  10. Dollar Sweets  11. Coca-Cola Australia  12. Sunshine Sugar  13. Rob Rees Food Works Australia Pty Ltd | 1. Australian Association of Convenience Stores  2. Ad Standards  3. Australian Beverages Council  4. Dairy Australia  5. Australian Association of National Advertisers  6. Australian Sugar Industry Alliance  7. Australian Food and Grocery Council  8. Free TV Australia  9. Australian Industry Group  10. Outdoor Media Association | 1. Brenda Janscheck (Health and Lifestyle)  2. Metabolic Health Solutions  3. The Root Cause  **4. Mashblox**  5. Johnson and Johnson Medical  **6. Medtronic**  **7 Novo Nordisk Pharmaceuticals Pty. Ltd** |
